# Fulminant course of unilateral emphysematous pyelonephritis revealing a renal actinomycosis caused by *Actinomyces meyeri*, an unknown cause of septic shock

**DOI:** 10.1186/2052-0492-2-42

**Published:** 2014-07-08

**Authors:** Alexandre Herbland, Maxime Leloup, Quentin Levrat, Frédéric Guillaume, Virginie Verrier, Philippe Bouillard, Thierry Landois, Charlie Frédéric Ouaki, Olivier Lesieur

**Affiliations:** Service de Réanimation Polyvalente, Hôpital Saint-Louis, 17019 La Rochelle, France; Service de Radiologie, Hôpital Saint-Louis, 17019 La Rochelle, France; Service d’Urologie, Hôpital Saint-Louis, 17019 La Rochelle, France

**Keywords:** Sepsis, Shock, *Actinomyces*, Actinomycosis, Emphysematous pyelonephritis, Pyuria

## Abstract

The objective of this case report is to describe the first case of renal actinomycosis caused by *Actinomyces meyeri* presenting as severe emphysematous pyelonephritis and complicated by septic shock and multi-organ failure. Emphysematous pyelonephritis is a potentially life-threatening infection mostly described in diabetic patients and predominantly caused by uropathogenic bacteria. Actinomycosis is an uncommon chronic infection due to anaerobic gram-positive bacteria that unusually involves the urinary tract. We report the first case of emphysematous pyelonephritis caused by *A. meyeri* in a 75-year-old non-diabetic woman. The patient presented with an altered status, fever, nausea, and vomiting lasting for 2 days. A computed tomography scan revealed unilateral emphysematous pyelonephritis. She was rapidly admitted to intensive care unit for a septic shock with multiple organ dysfunctions. A conservative management consisting in renal percutaneous drainage, supportive measures, and prolonged adapted antibiotic therapy resulted in complete recovery. This case report illustrates that renal actinomycosis should be considered in case of emphysematous pyelonephritis given the good prognosis of this infection with conservative medical treatment.

## Background

Emphysematous pyelonephritis (EPN) is a potentially life-threatening disease characterized by a severe necrosis of the renal parenchyma with presence of gas in the renal parenchyma, collecting system, and/or perinephric tissue. *Escherichia coli* is the commonest causative organism found for about 70% of cases. Other organisms like *Proteus*, *Pseudomonas*, *Klebsiella*, and *Acinetobacter* can also be found [1]. Actinomycosis is an uncommon chronic infection due to a gram-positive anaerobic bacterium. *Actinomyces israelii* is the most common human pathogen among all *Actinomyces* pathogenic species. The cervicofacial area, pelvic region, and thoracic involvement are the most common clinical presentations, but very few cases of renal actinomycosis (RA) are reported. Our literature review uncovered only one prior report of EPN revealing a RA after nephrectomy [2]. We report the first case of RA with *Actinomyces meyeri* in urine cultures presenting as an EPN complicated by a septic shock and successfully treated with conservative management.

## Case presentation

A 75-year-old female presented to a local clinic with a history of fever, nausea, vomiting, and anorexia lasting for 2 days. Medical history was significant only for arterial hypertension, obesity (body mass index 29), and urinary incontinence. She was treated by oral antihypertensive therapy and solifenacin for her overactive bladder.

**Figure 1:**
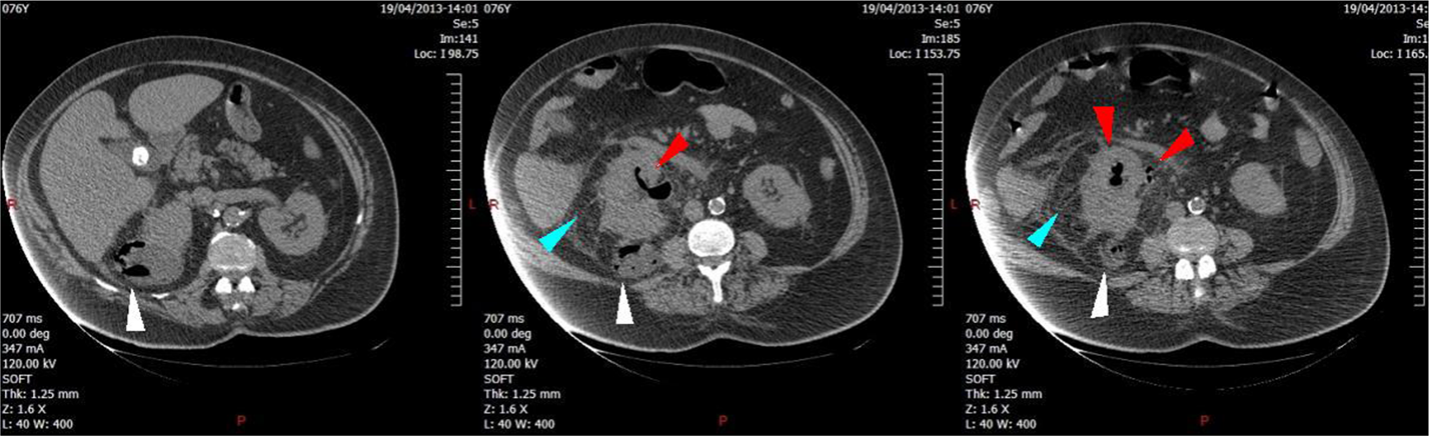
**Emphysematous pyelonephritis (EPN) of the right kidney.** Computed tomography scan demonstrates right-sided EPN with enlarged right kidney and normal left kidney. Gas is present in the renal pelvis, in the proximal ureter (red arrows), and in a posterior cyst of the right kidney (white arrows). There is also significant perirenal infiltration (blue arrows).

Physical examination revealed fever with body temperature of 101.3°F (38.5°C), tachypnea with respiratory rate of 25 breaths/min, normal hemodynamic parameters with blood pressure 125/57 mmHg, and pulse 80 beats/minute. The laboratory investigations yielded an inflammatory process and moderate renal impairment. The white blood cell count was 37.6 × 10^9^/L with neutrophils 85%. Hemoglobin was 100.2 g/L. The platelet count was normal 263 × 10^9^/L, and fibrinogen was 32.89 μmol/L. C-reactive protein was 591 mg/L; the serum gammaglutamyltransferases level was increased up to 276 U/L. The alkaline phosphatases level was 168 U/L. The bilirubin was 9.7 μmol/L. The urea nitrogen and creatinine serum levels were, respectively, 18 mmol/L and 282 μmol/L. The other laboratory tests for blood chemistry panel and liver function were normal. Few minutes after her admission, the patient's blood pressure dropped dramatically with anuria and hypoxemia, so she received oxygen therapy (10 L/min with a reservoir mask) and intravenous fluid therapy with 1,500 mL of crystalloid (Ringer lactate) and 500 mL of colloid (6% hydroxyethyl starch 130/0.4).Urgent abdominal computed tomography (CT) scan permitted the diagnosis of EPN showing a pyonephrosis of the right kidney and presence of pneumaturia in the collecting system and in a cyst located in the lower pole of the right kidney (Figure 1). The pelvi-caliceal cavities of the right kidney appeared slightly dilated. After blood cultures, intravenous antibiotic therapy was initiated with amoxicillin/clavulanic acid 1 g and ofloxacin 200 mg. Despite antibiotic therapy and supportive measures, the patient's clinical condition rapidly deteriorated towards a septic shock with multiple organ dysfunction syndrome. The patient required mechanical ventilator support and intravenous vasopressor (norepinephrine). She was promptly transferred to our hospital to undergo urgent ureteral stenting.

On admission to the operating room, protective ventilation settings (controlled mandatory ventilation, tidal volume of 7 mL/kg of predicted body weight, respiratory rate 20 breaths/min, FiO_2_ 1.0) resulted in a PaO_2_/FiO_2_ ratio at 122. Hemodynamic parameters with continuous intravenous norepinephrine 0.4 gamma/kg/min showed pulse 90 beats/min and mean arterial pressure 69 mmHg, and the patient was still anuric. A right retrograde cystoscopic pyelogram revealed a moderate pelvi-caliceal dilatation and also allowed a right ureteral open-ended stenting with drainage of a pyuria.

The patient was subsequently admitted to our ICU. Antibiotics were switched to intravenous ceftriaxone (2 g/day) plus ofloxacin (200 mg twice a day) and metronidazole (500 mg every 8 h). Septic shock improved in the following 2 days. Her renal function recovered without need of renal replacement therapy and with equal diuresis of both kidneys. Despite improvement of the patient's condition, we observed a reascension of WBC at day 3 (50 × 10^9^/L). Gynecological examination with hysteroscopy excluded genital infection. CT scan showed a persistent posterior abscess of the right kidney with inflammatory infiltration of the perirenal space. After discussion with urologists and radiologists, a percutaneous CT scan-guided drainage was performed by a radiologist who placed a stent in the abscess and collected a hemic and purulent fluid. The patient's condition rapidly improved, so she was extubated on day 8. On day 11, the CT scan-guided drainage was complicated by a fistula, with the urinary tract successfully treated by a double J stenting of the right ureter.

**Figure 2:**
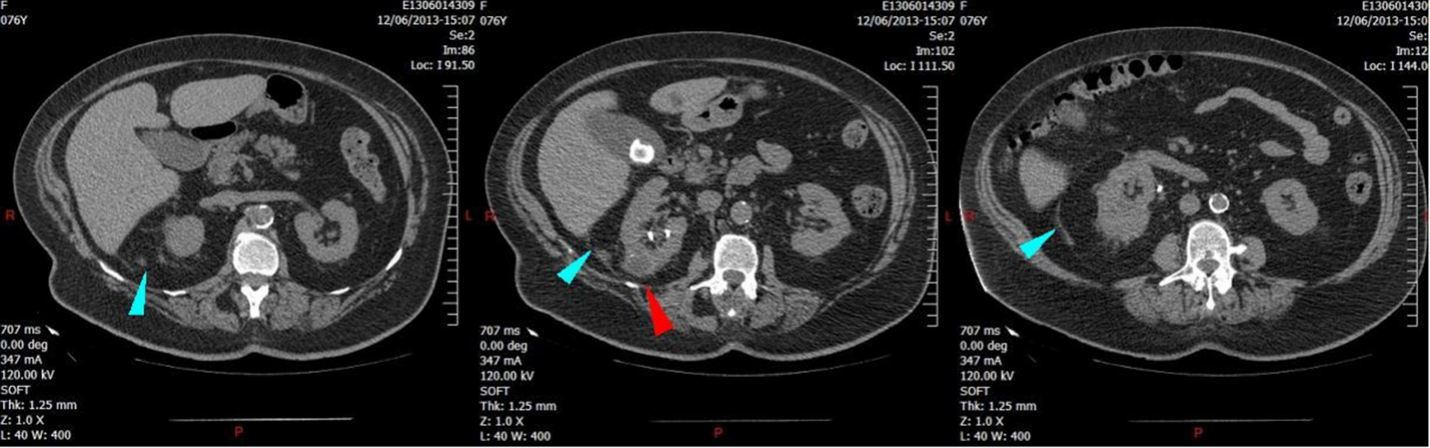
**The 2-month follow-up computed tomography scan.** The follow-up computed tomography scan shows the normalizing size and complete absence of gas in the right kidney. A small posterior cyst of the right kidney is still present (red arrow). Perirenal infiltration has almost disappeared (blue arrows).

Microscopic examination of urine collected by cystoscopy from the right pelvi-caliceal cavity showed presence of pyuria, Gram stain detected gram-positive bacilli, and culture was positive with *A. meyeri* 10^5^ CFU/mL with the use of Vitek 2 ANC card (bioMérieux, Marcy l'Etoile, France). Antibiotherapy was finally switched to amoxicillin 2 g × 3 per day according to the antibiogram for a duration of 3 months against *A. meyeri*. As advised by our infectious disease specialists, ofloxacin (200 mg twice a day) was maintained for 21 days covering a hypothetic non-diagnosed coinfection with gram-negative bacilli. Metronidazole was stopped at day 5 after identification of *A. meyeri*. At day 10, the patient was finally discharged from the ICU to the department of urology in stable condition. She recovered a normal renal function and was transferred back to her home at day 24. She underwent an abdominal CT scan 2 months later (Figure 2) showing regression of the right pyonephrosis with normal parenchyma, no dilatation, and persistence of a right renal cyst of reduced diameter. Four months later, ureteroscopic examination revealed no abnormality and double J stenting was retrieved.

## Discussion

EPN is a renal infection caused predominantly by gram-negative rods which are capable of fermenting glucose in an anaerobic environment [3]. *E. coli* is the commonest organism found in about 75% of patients with EPN; other organisms like *Klebsiella* and *Proteus* are also reported [1]. EPN caused by various fungi like *Candida* and *Aspergillus fumigatus* are also sporadically documented [4–6]. EPN predominantly concerns women (female/male ratio is 6:1) and can be unilateral or bilateral [1, 7, 8]. Around 95% of patients developing EPN have uncontrolled diabetes mellitus (DM), but other associated factors are reported as drug abuse, neurogenic bladder, alcoholism, immune-compromised status, anatomical anomaly, or presence of urinary tract obstruction [1, 7–10]. In our case, DM was not present, but the patient had two associated risks for EPN: urinary incontinence and a cyst on the right kidney, an anatomical particularity which may have induced local urine retention with moderate obstruction of the pelvi-caliceal cavities.

Diagnosis was made by CT scan which is currently the radiographic method of choice both for diagnosing EPN and demonstrating the extent of the disease [1]. Sonographic diagnosis of EPN is also possible by the recognition of echogenic foci with ‘dirty’ shadowing in a non-dependent position, highly suggestive of gas inside the kidney [11]. Ultrasonography can also be useful to realize guided percutaneous drainage of renal abscesses. But the presence of gas within the renal parenchyma in EPN produces artifactual reverberation echoes, compromising the quality of the ultrasound images. Thus, we opted for CT scan-guided percutaneous drainage of the renal abscess. Moreover, CT scan allows much more accuracy than ultrasonography for the drain placement because the entire guidewire or drainage catheter is usually demonstrated more clearly by CT than by ultrasound. In patients with EPN, beside aggressive medical management (prompt appropriate antibiotics and resuscitation measures with multi-organ support), percutaneous drainage seems to be the most successful management compared with medical management alone or plus nephrectomy with the lowest mortality at 13.5% (*P* < 0.001) [12].

Actinomycosis is a rare chronic infection due to anaerobic gram-positive bacteria that unlikely involves the urinary tract [13–15]. This infection is due to a group of bacteria which are normal commensals found in the mouth and gastrointestinal tract, with *A. israelii* being the most common agent identified in human infections and particularly in RA. It seems that *Actinomyces* are able to invade mucous membranes under certain circumstances such as mucosal alteration secondary to trauma or infection by other organisms [16]. Poor hygiene, underlying diseases, or immunosuppression can be found in patients with actinomycosis, but predisposing factors are not systematically documented [17]. In our case, it is still unclear how the patient contracted the infection, as there was neither history of previous surgical treatment nor other risk factors identified. Hematogenous origin can be hypothesized without certainty.

*Actinomyces meyeri* was first isolated from a patient with a lung empyema by Meyer in 1911, who termed it *Streptothrix*. The nomenclature was changed to *Actinobacterium meyerii* by Prevot in 1956 and subsequently to *Actinomyces meyeri* by Holderman in 1977 [18]. A recent review of the literature revealed only 32 documented cases of infection with *A. meyeri* [19] *.* According to this article, 13 patients had a disseminated disease, and the most frequent sites of infection were pulmonary, gastrointestinal, skin, and soft tissues, respectively. No case of urinary tract infection was reported.

The few cases of RA published are characterized by a medical presentation mimicking malignancy with pseudo-tumoral aspect, insidious course, and non-specific symptoms [20–22]. The diagnosis is difficult, and only few patients have avoided nephrectomy or at least surgical exploration. Diagnosis can be made by ultrasound or CT-guided fine needle biopsy [23, 24]. In our particular case, microbiological diagnosis of actinomycosis was based on concordant Gram stain result (gram-positive bacilli) and positive urine culture collected in the upper urinary tract under ureteroscopy. As we opted for a non-invasive treatment, we did not perform any biopsy for histological examination. Furthermore, other urine and blood cultures (including those taken before first antibiotherapy) failed to grow any other microorganism.

A case of RA presenting as EPN and complicated by septic shock was already reported from Taiwan [2]. The patient recovered after medical treatment and nephrectomy. Diagnostic of RA was made on histological examination of the surgical kidney and ureteral specimens. Microbiological identification of *Actinomyces* species is not detailed in the article, and the patient had a urinary tract coinfection with *E. coli* that could explain such an emphysematous presentation. Our search for published literature did not reveal any report of urinary tract infection or EPN caused by *A. meyeri*.

*Actinomyces* are usually susceptible to a wide variety of antimicrobial agents including penicillin G, cephalosporins, tetracycline, erythromycin, clindamycin, imipenem, and streptomycin [25, 26] but are resistant to fluoroquinolones which are widely prescribed for urinary tract infections. There is no consensus recommendation for medical treatment of RA and no particular sensitivity or resistance to antibiotics identified for *A. meyeri* given the small number of cases reported [19]. Clinical experience supports the use of intravenous penicillin G as the drug of choice (18 to 24 million U/day) for 2 to 6 weeks followed by oral therapy. For other *Actinomyces* infections, a prolonged duration of antibiotic therapy is advised to avoid relapse varying to 2 months for the localized disease to 12 months for more extensive and disseminated disease [26]. The treatment needs to be individualized based on the clinical and radiological response.

## Conclusions

RA is a rare and difficult infection to recognize and should be considered of etiological diagnosis for EPN. This first case report of severe EPN secondary to RA caused by *A. meyeri* clearly illustrates that EPN is a potentially life-threatening disease. A CT scan-guided percutaneous drainage of renal abscess was a safe and effective procedure which permitted decisive improvement of the patient's condition. Awareness of this entity may encourage physicians to make the diagnosis and to implement effective long-lasting antibiotic therapy, avoiding futile surgery and preserving further renal function.

## Consent

Written informed consent was obtained from the patient for publication of this case report and any accompanying images. A copy of the written consent is available for review by the Editor-in-Chief of this journal.
